# Inhibition of Notch1 Signaling Alleviates Endotoxin-Induced Inflammation Through Modulating Retinal Microglia Polarization

**DOI:** 10.3389/fimmu.2019.00389

**Published:** 2019-03-08

**Authors:** Zhixing Cheng, Yao Yang, Fang Duan, Bingsheng Lou, Jieting Zeng, Yanqiao Huang, Yan Luo, Xiaofeng Lin

**Affiliations:** State Key Laboratory of Ophthalmology, Zhongshan Ophthalmic Center, Sun Yat-Sen University, Guangzhou, China

**Keywords:** Notch1 signaling, retinal microglia, polarization, endotoxin-induced uveitis, DAPT, inflammation

## Abstract

Microglial cells are resident immune cells and play an important role in various cerebral and retinal inflammatory diseases. Notch1 signaling is involved in the microglia polarization and the control of cerebral inflammatory reactions. However, its role in endotoxin-induced uveitis (EIU) remains unknown. This study aimed to investigate the role of Notch1 signaling on retinal microglia polarization and inflammation in the cultured retinal microglial cells and EIU rat model. We found that Notch1 signaling blockade with N-[N-(3, 5-difluorophenacetyl)-1-alany1-S-phenyglycine t-butyl ester (DAPT) shifted retinal microglia phenotype from pro-inflammatory M1 phenotype (COX2^+^ and iNOS^+^) to anti-inflammatory M2 phenotype (Arg-1^+^) and reduced the release of pro-inflammatory cytokines both *in vivo* and *in vitro*. Moreover, DAPT treatment contributed to prevent retinal ganglion cells from apoptosis, reduce the intraocular infiltrating cells, and attenuate the impairment of retinal function. Taken together, these results suggest that inhibition of Notch1 signaling could alleviate the inflammatory response in EIU rat mainly through regulating the polarization of retinal microglia. Therefore, Notch1 signaling might be a promising therapeutic target in the treatment of ocular inflammatory diseases.

## Introduction

Uveitis composes a group of diseases characterized by intraocular inflammation and is a major cause of blindness worldwide (1). The severe and irreversible visual loss usually ascribes to retinal damage caused by amplification and over-reaction of inflammation (2). The current main therapies for uveitis contain the systemic administration of corticosteroid and immunosuppressant. However, the treatment effect is not always satisfactory and the long time use of these agents often results in severe side effects, such as glaucoma, cataract, and the impairment of liver and kidney (3). Therefore, it is essential to find some novel treatment approaches for uveitis to avoid irreversible visual loss and corticosteroid and immunosuppressant-related complications.

Microglial cells, as the resident immune cells, constitute the first defensive line in the healthy brain and retina (4). Microglia activation is a common hallmark of various inflammatory diseases and the microglia-mediated inflammation often leads to neuronal damage (5). Depending on the condition, activated microglia may acquire two different kinds of polarization: pro-inflammatory M1 phenotype and anti-inflammatory M2 phenotype (6–9). The activated M1 microglial cells constantly release pro-inflammatory cytokines to induce microglia over-activation and uncontrolled inflammatory response and damage tissues. Whereas, the activated M2 microglial cells can release anti-inflammatory and neuroprotective factors to promote tissue repair. Thus, modulating microglia polarization emerges as a promising therapeutic strategy for inflammatory diseases. Recent studies suggest that Notch1 signaling is involved in regulating microglia activation and polarization and controlling inflammation in various cerebral diseases (10–16). However, the role of Notch1 signaling in retinal microglia polarization and inflammation of uveitis is still unknown. Endotoxin-induced uveitis (EIU) rat model is a classical animal model, which is widely used in the study of acute intraocular inflammation (17). Therefore, this study aimed to investigate the role of Notch1 signaling on retinal microglia polarization and inflammation in the primary cultured retinal microglia and EIU rats.

## Materials and Methods

### Primary Culture and Treatment of Retinal Microglia

The primary retinal microglia culture was prepared from postnatal day one (P1) male or female Sprague-Dawley (SD) rats' retinas as previously described (18). Retinas of both eyes from 18 postnatal SD rats were used to make the culture of retinal microglial cells each time. The retinas were carefully removed, put in the centrifuge tube, and blown into single cell suspensions by pipette. The cells were seeded in a 75 cm^2^ culture flask and cultured in DMEM/F12 with 20% fetal bovine serum, 100 U/mL penicillin, and 100 μg/mL streptomycin. The culture medium was replaced completely at the second day and then added 3 ml every 3 days. When the cultured cells reached confluence at 10–14 days, retinal microglial cells were isolated from the mixed glial cells by shaking on a rotary shaker at 175 rpm for 1 h and further purified by differential adhesion method for 30 min. The ionized calcium-binding adapter molecule 1 (Iba1), a specific marker of microglia, was used to identify retinal microglial cells. Retinal microglial cells with more than 96% purity were seeded on 6-well-plates and used for experiment. Retinal microglial cells were treated with 10 ng/mL lipopolysaccharide (LPS) (Sigma-Aldrich, USA) for 6 and 12 h. N-[N-(3, 5-difluorophenacetyl)-1-alany1-S-phenyglycine t-butyl ester (DAPT) (Sigma-Aldrich, USA), a novel γ-secretase inhibitor, was used to inhibit Notch1 signaling at a final concentration 20 μM 1 h before LPS stimulation. Retinal microglial cells were randomly divided into three groups: Control group treated with sterile phosphate buffer solution (PBS), LPS group treated with LPS, and DAPT+LPS group treated with DAPT and LPS. The cells in one well of 6-well-plates were harvested at 6 and 12 h after LPS stimulation for further examinations.

### EIU Rat Model and Treatment

Eight-week-old male SD rats (200–250 g) were randomly divided into three groups: Control group, LPS group and DAPT+LPS group. The rat right eyes in each group were intravitreally injected with sterile PBS (Control group), LPS (LPS group), and DAPT and LPS (DAPT+LPS group), respectively. All animal studies were performed in accordance with the Association Research in Vision and Ophthalmology (ARVO) resolution on the use of animals in research and approved by the Zhongshan Ophthalmic Center Animal Care and Use Committee, Sun Yat-sen University, Guangzhou, China (authorized number 2016-141). EIU rat model was induced by a single intravitreal injection with 2 μl of 125 ng/μl LPS. One hour before LPS injection, 1 μl of 10 mM DAPT was intravitreally injected to block Notch1 signaling. SD rats in each group were sacrificed and the right eyes were enucleated at 6, 12, and 24 h after LPS stimulation for further analysis.

### Quantitative Real-Time PCR (qRT-PCR)

Total RNA was isolated from the primary cultured retinal microglia in one well of 6-well-plates (*n* = 3 in each group) using the RNAiso Plus (Takara, Japan) and then reversely transcribed to cDNA using PrimeScript™RT reagent kit (Takara, Japan). The nucleic acid purity was quantified and analyzed using spectrophotometry (NanoDrop Technologies, Wilmington, DE). Primers (Table 1) were designed using Primer Premier 5.0 software (PREMIER Biosoft International, Palo Alto, CA). Gene expression levels were measured by the LightCycler 480 system (Roche). The PCR procedure was as follows: pre-incubation for 5 min at 95°C, followed by 40 cycles amplification of denaturation for 10 s at 95°C and annealing for 15 s at 60°C. The reactions of each cDNA sample were performed in triplicate. The expression level of each gene was expressed as fold expression after normalized to the reference gene (GAPDH).

**Table 1:** Primers used for qRT-PCR.

**Gene**		**Primers**
Notch1	Forward	CGT GCT ATG TTG TGG ACC ATG GC
	Reverse	CAC ACT CGT GGG TGG TGT CCC CCG
iNOS	Forward	GAC CAG AAA CTG TCT CAC CTG
	Reverse	CGA ACA TCG AAC GTC TCA CA
COX2	Forward	AGT ATC AGA ACC GCA TTG CC
	Reverse	TAA GGT TTC AGG GAG AAG CG
Arg-1	Forward	TGC CGT GTT CAC AGT ACG AGT C
	Reverse	AAG GAA GAA AAG GCC CAT TCA
Hes1	Forward	GTC CCG CTG TTG CTG GTG TAG
	Reverse	GAC GGC CAA TTT GCT TTC CTC
GAPDH	Forward	GGA TGG AAT TGT GAG GGA GA
	Reverse	GTG GAC CTC ATG GCC TAC AT

### Western Blotting

The total protein was extracted from the cultured retinal microglia in one well of 6-well plates (*n* = 3 in each group) or rat retinas (*n* = 4 in each group) with lysis buffer (KeyGen, China) containing protease and phosphatase inhibitor. The protein concentration was measured using Pierce™ BCA Protein Assay Kit (Thermo Scientific, USA). Equal amount of protein from each sample was subjected to 8–10% sodium dodecyl sulfate-polyacrylamide gels, and then transferred to polyvinylidene difluoride membranes (Bio-Rad, USA). The membranes were incubated with primary antibodies against Notch1 (Cat. 4380, CST), Notch intracellular domain (NICD) (Lot.GR317746-16, ab52301), iNOS (Cat. PA1-036, Thermofisher), COX2 (Cat. 12282, CST), Arg-1 (Cat. 93668, CST), Hes1 (Cat. 11988, CST), and β-tubulin (Cat. 2128, CST) overnight at 4°C. The membranes were then incubated with secondary antibodies (ab6802, abcam) for 1 h. Protein bands were visualized using the ChemiDoc Touch Imaging System (Bio-Rad, USA). The band intensity was quantified using Image J software (NIH).

### ELISA

Vitreous samples were prepared according to a previous study (19). The cell supernatant and vitreous humor from four right eyes in each group pooled as one sample (*n* = 12 rats in each group) were collected 12 h after LPS stimulation and stored at −80°C for further use. The concentrations of the inflammatory cytokines, such as TNF-α (Cat. ELR-TNFα-CL, RayBiotech, USA), IL-6 (Cat. ELR-IL6-CL, RayBiotech, USA), and IL-1β (Cat. ELR-IL1b-CL, RayBiotech, USA), in the cell supernatant and vitreous humor of each group were measured with ELISA kits following the manufacturer's instructions.

### Electroretinogram (ERG)

ERG recordings of rats (*n* = 6 in each group) were performed with RETI-scan system (Roland Consult, Germany) at a sampling rate of 2 kHz 24 h after injection. All experimental rats underwent a dark adaptation for 12 h prior to the daytime tests. SD rats were anesthetized with 10% chloral hydrate (3 ml/kg) through intraperitoneal injection. Pupils were dilated with Tropicamide Phenylephrine eye drops and corneas were anesthetized with 0.5% tetracaine hydrochloride eye drops. ERG was recorded with a gold-plated wire loop electrode contacting the corneal surface as an active electrode. Stainless steel needles ripped into the skin near the eye and into the tail as the reference and ground electrode, respectively. The amplitudes of a-wave and b-wave were recorded as the average of three responses under 0.3 and 3.0 cd·s/m^2^ flash stimuli intensities.

### Immunofluorescence Assay on Retinal Flat Mounts

The right eyes (*n* = 3 in each group) were enucleated and fixed in 4% paraformaldehyde for 30 min. Retinas were prepared carefully and incubated with primary antibody against Iba1 (ab178847, abcam) for 48 h and washed in PBST, and then incubated with secondary antibody conjugated with Alexa Fluor®488 (ab150073, abcam). After washed with PBST, retinas were mounted with anti-fade mounting medium and images were collected by a confocal microscope (Carl Zeiss LSM710, Germany). Three images were randomly captured in the central area (~1 diameter of optic disc distant from margin of the optic nerve head) of each retina.

### Histopathological Analysis

The enucleated eyes (*n* = 3 in each group) were fixed in 4% formalin for 24 h, then washed with PBS and dehydrated using the gradient reagent alcohol, and then embedded in paraffin. 5 μm of rat eye sections through optic disc were cut, deparaffinized, and stained with hematoxylin and eosin (H&E) for histopathologic analysis of uveitis symptoms. Intraocular inflammatory cells were calculated to assess the severity of uveitis symptoms. The sections throughout optic disc were photographed with a microscope (Leica DM4000, Germany). Three images in each eye were randomly captured in a field center on optic disc at a final magnification of 200× for counting inflammatory cells. The counting of inflammatory cells was performed by two experienced researchers.

### TUNEL Assay

Retinas (*n* = 3 in each group) were embedded in optimal cutting temperature compound (Sakura Finetechnical Co., Japan) at −80°C, and sectioned through optic disc to a thickness of 14 μm retinal frozen sections. The apoptotic assay was performed on the retinal frozen sections using TUNEL kit (*in situ* Cell Death Detection Kit, TMR red, version 12, Roche, USA) according to the manufacturer's instructions. The retinal sections were then stained with DAPI and photographed using a confocal microscope (Carl Zeiss LSM710, Germany). Three images were randomly captured in the center area of each retina at a final magnification of 200×. The number of TUNEL-positive cells in each retinal frozen section was counted by two experienced researchers.

### Statistical Analysis

All the *in vitro* experiments were performed in triplicate wells at each time and the rat numbers of each *in vivo* experiment were ranged from 3 to 12. Each experiment in our study was repeated independently with three different batches of cells or rats. Statistical analysis was performed using SPSS software 17.0 (IBM, USA). The mean±SEM values comparisons of multiple groups were analyzed using one-way analysis of variance (ANOVA) followed by Tukey's *post hoc* test. A value of *p* < 0.05 was considered significant.

## Results

### Notch1 Signaling in the Cultured Retinal Microglia Was Activated by LPS and Blocked by DAPT

**Figure 1:**
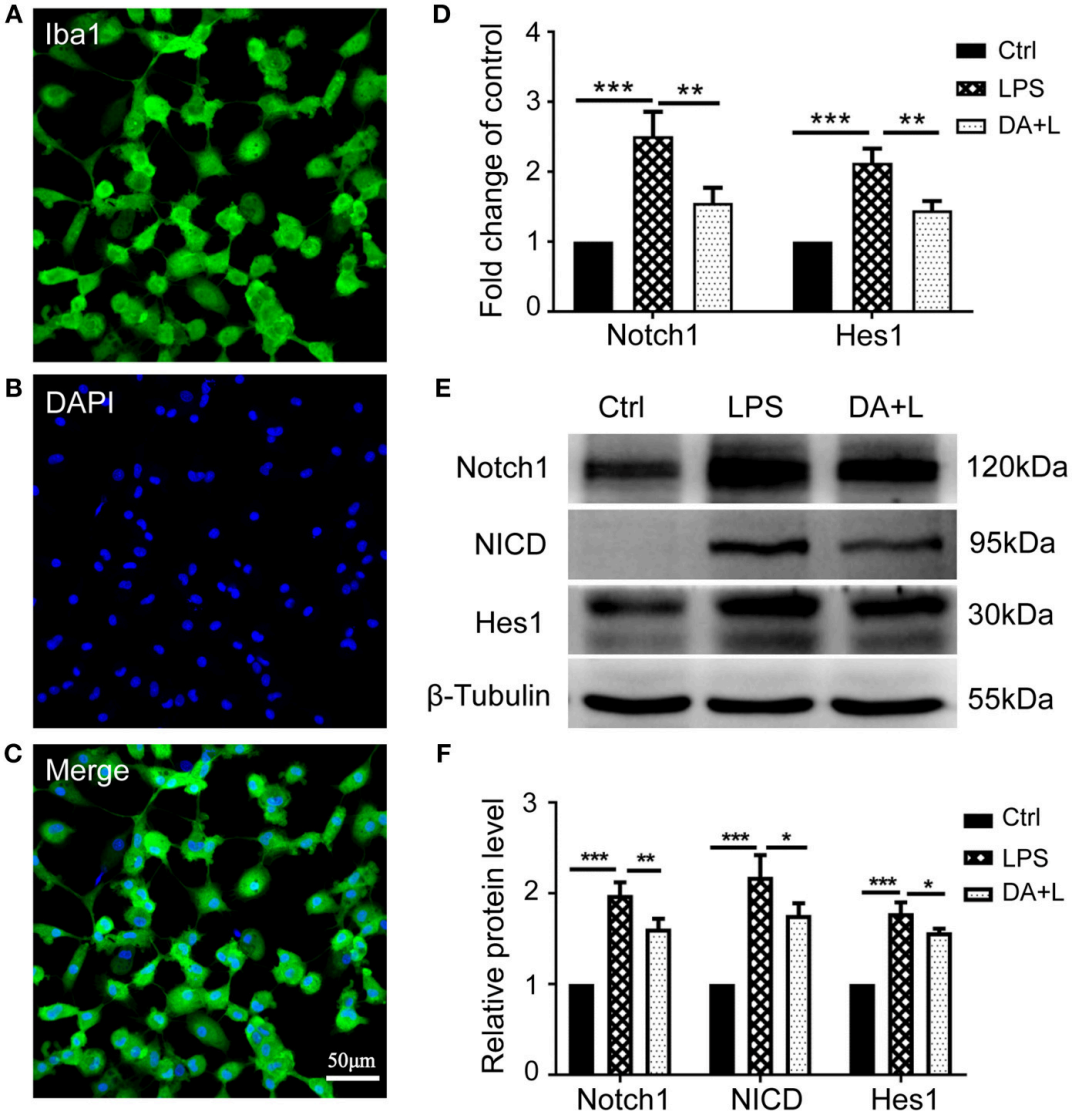
Notch1 signaling was activated by LPS and inhibited by DAPT in primary cultured retinal microglia. **(A–C)** Confocal microscope images showed that more than 96% cultured cell were positively stained with Iba1 (green), and DAPI showing nuclei (blue). Scale bar, 50 μm. **(D)**The mRNA expressions of Notch1 and Hes1 were significantly upregulated after LPS stimulation compared with the control. DAPT pretreatment reduced the increased expressions of Notch1 and Hes1 induced by LPS. **(E,F)** The protein expressions of Notch1 and Hes1 were consistent with the results of mRNA expression. In addition, DAPT treatment can suppress the increased protein expression of NICD induced by LPS. *p < 0.05, **p < 0.01, and ***p < 0.001 (one-way ANOVA). Ctrl, Control; DA+L, DAPT+LPS.

To investigate the purity of primary cultured retinal microglial cells, microglial cells were identified using Iba1. The retinal microglial cells presented a highly ramified morphology in resting state and more than 96% cultured cells were positively stained with Iba1 (Figures 1A–C), meeting the experimental requirements. After retinal microglial cells were challenged with LPS for 6 and 12 h, both the mRNA and protein levels of Notch1 receptor were significantly upregulated compared with the control. As the main downstream target gene of Notch1 signaling, the mRNA and protein levels of Hairy enhancer of split-1 (Hes1) concurrently increased compared with the control. DAPT pretreatment could remarkably suppress the increased expression of Notch1 and Hes1 induced by LPS. Furthermore, the protein level of NICD which indicates an increase in the activation of Notch1 signaling was induced by LPS stimulation and suppressed by DAPT treatment. These results demonstrated that Notch1 signaling in retinal microglia could be effectively activated by LPS and blocked by DAPT (Figures 1D–F).

### Notch1 Signaling Modulated Retinal Microglia Polarization *in vitro*

**Figure 2:**
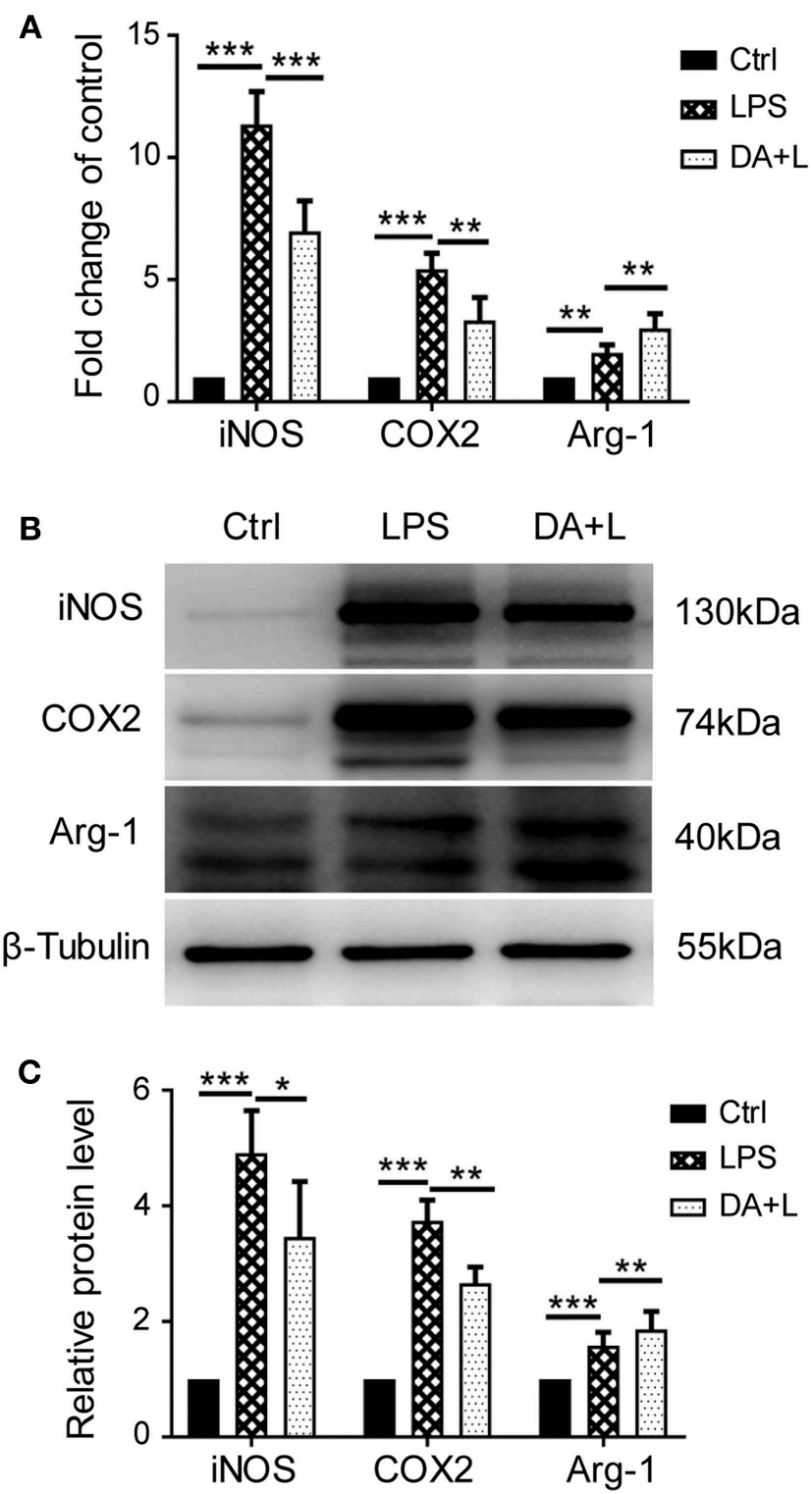
Notch1 signaling regulated retinal microglia polarization *in vitro*. **(A)** The mRNA expressions of M1 phenotype-specific markers (iNOS and COX2) and M2 phenotype-specific marker (Arg-1) were markedly increased in the primary cultured retinal microglia by LPS stimulation compared with the control. DAPT pretreatment suppressed the expressions of iNOS and COX2, but further enhanced Arg-1 expression. **(B,C)** The protein expressions of iNOS, COX2 and Arg-1 were consistent with the results of mRNA expression. *p < 0.05, **p < 0.01, and ***p < 0.001 (one-way ANOVA). Ctrl, Control; DA+L, DAPT+LPS.

Depending on different factors received by the microglia receptors, activated microglial cells appear as a classic M1 phenotype or an alternatively activated M2 phenotype. In order to investigate how Notch1 signaling modulates the retinal microglia polarization, the protein and mRNA levels of phenotype-specific markers including iNOS, COX2, and Arg-1 were measured. The protein and mRNA expression of iNOS and COX2 (M1 specific markers) and Arg-1 (M2 specific marker) were significantly upregulated in retinal microglia challenged with LPS. DAPT pretreatment could markedly suppress the increased expression of iNOS and COX2, but further enhanced the expression level of Arg-1 (Figures 2A–C). These results suggested that inhibition of Notch1 signaling could suppress the M1 phenotype microglia and drive activated microglia toward the M2 phenotype.

### Notch1 Signaling Blockade Reduced the Production of Inflammatory Cytokines *in vitro*

**Figure 3:**
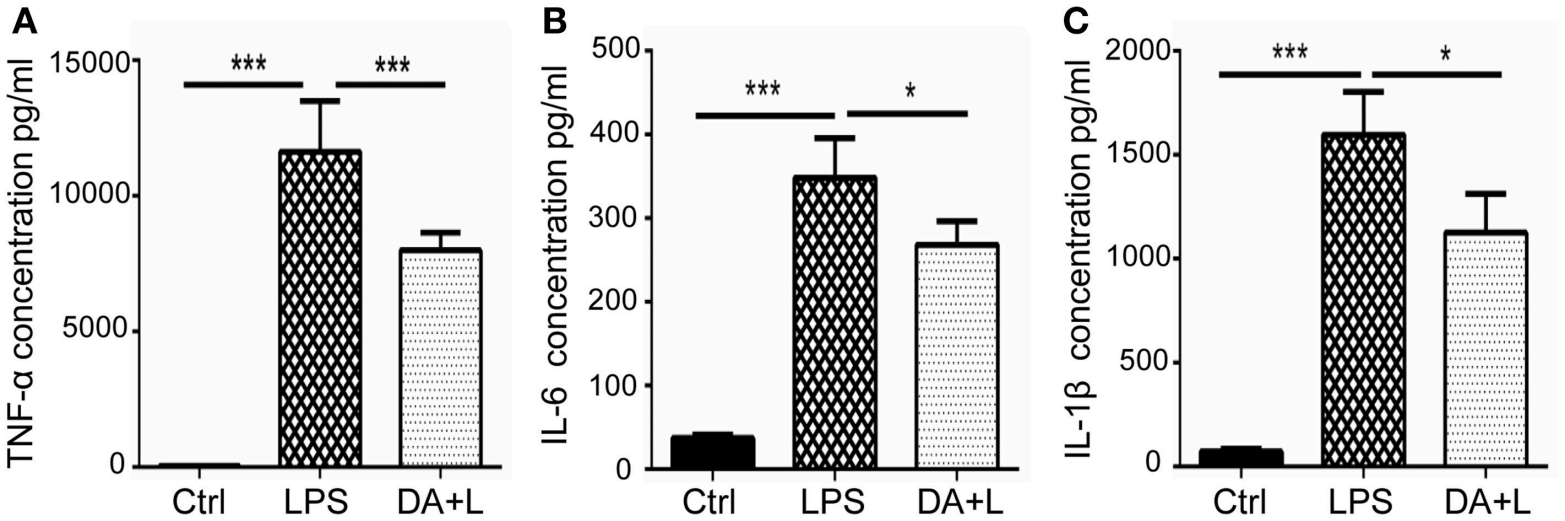
Notch1 signaling blockade reduced the production of inflammatory cytokines *in vitro*. **(A–C)** ELISA results showed that the concentrations of TNF-α **(A)**, IL-6 **(B)**, and IL-1β **(C)** were significantly increased after LPS stimulation in cell supernatant compared with the control. DAPT pretreatment reduced the release of TNF-α, IL-6 and IL-1β. *p < 0.05, ***p < 0.001 (one-way ANOVA). Ctrl, Control; DA+L, DAPT+LPS.

As the increased expression of inflammatory cytokine is considered as the hallmark of activated microglia, we next investigated whether inhibition of Notch1 signaling could affect the secretion of inflammatory cytokines by activated retinal microglial cells. ELISA results showed that concentrations of TNF-α, IL-6, and IL-1β in cell supernatant were significantly increased after LPS stimulation compared with the control. DAPT treatment reduced the release of TNF-α, IL-6, and IL-1β induced by LPS *in vitro* (Figures 3A–C).

### DAPT Treatment Attenuated the Impairment of Retinal Function Induced by LPS

**Figure 4:**
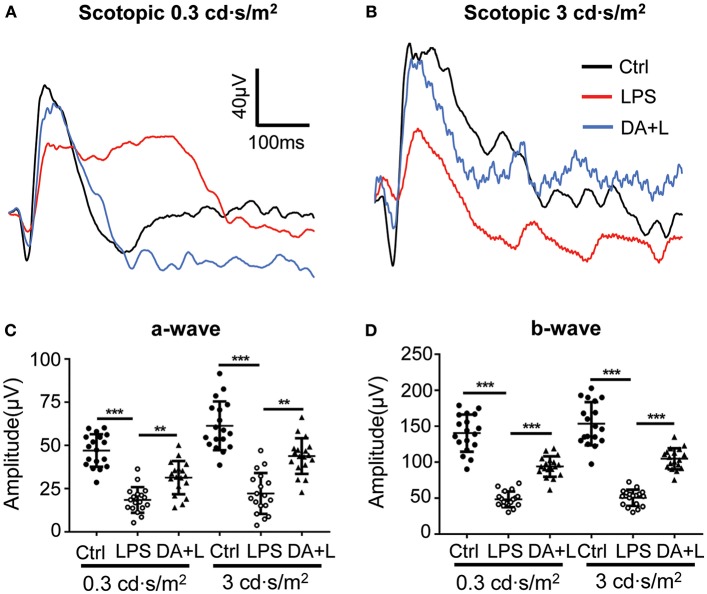
DAPT treatment attenuated the impairment of the retinal function induced by LPS. **(A,B)** Retinal function was evaluated by flash ERG under two light intensities. The amplitudes of a-wave and b-wave were significantly decreased 24 h after LPS injection under two different stimuli intensities compared with those in the control group. However, DAPT treatment could markedly improve the amplitudes of a- and b-wave compared with that in the LPS group. Total 18 rats in each group were used for statistical analysis. **(C,D)** The dot plots of a-wave and b-wave under two light intensities. **p < 0.01, ***p < 0.001 (one-way ANOVA). Ctrl, Control; DA+L, DAPT+LPS.

LPS-induced uveitis had significant influence on the retinal neurons and function. ERG has been an objective tool to assess the retinal function. In order to evaluate the retinal damage induced by LPS and the protective efficacy of DAPT, we used flash ERG to evaluate the retinal function in each group. The amplitudes of a-wave and b-wave were significantly decreased 24 h after LPS injection compared with that of the control group. However, DAPT treatment could markedly improve amplitudes of a-wave and b-wave compared with that of the LPS group (*p* < 0.05) (Figures 4A–D). These results indicated that DAPT treatment could attenuate the impairment of retinal function induced by LPS.

### Notch1 Signaling Modulated the Activation and Polarization of Retinal Microglia *in vivo*

**Figure 5:**
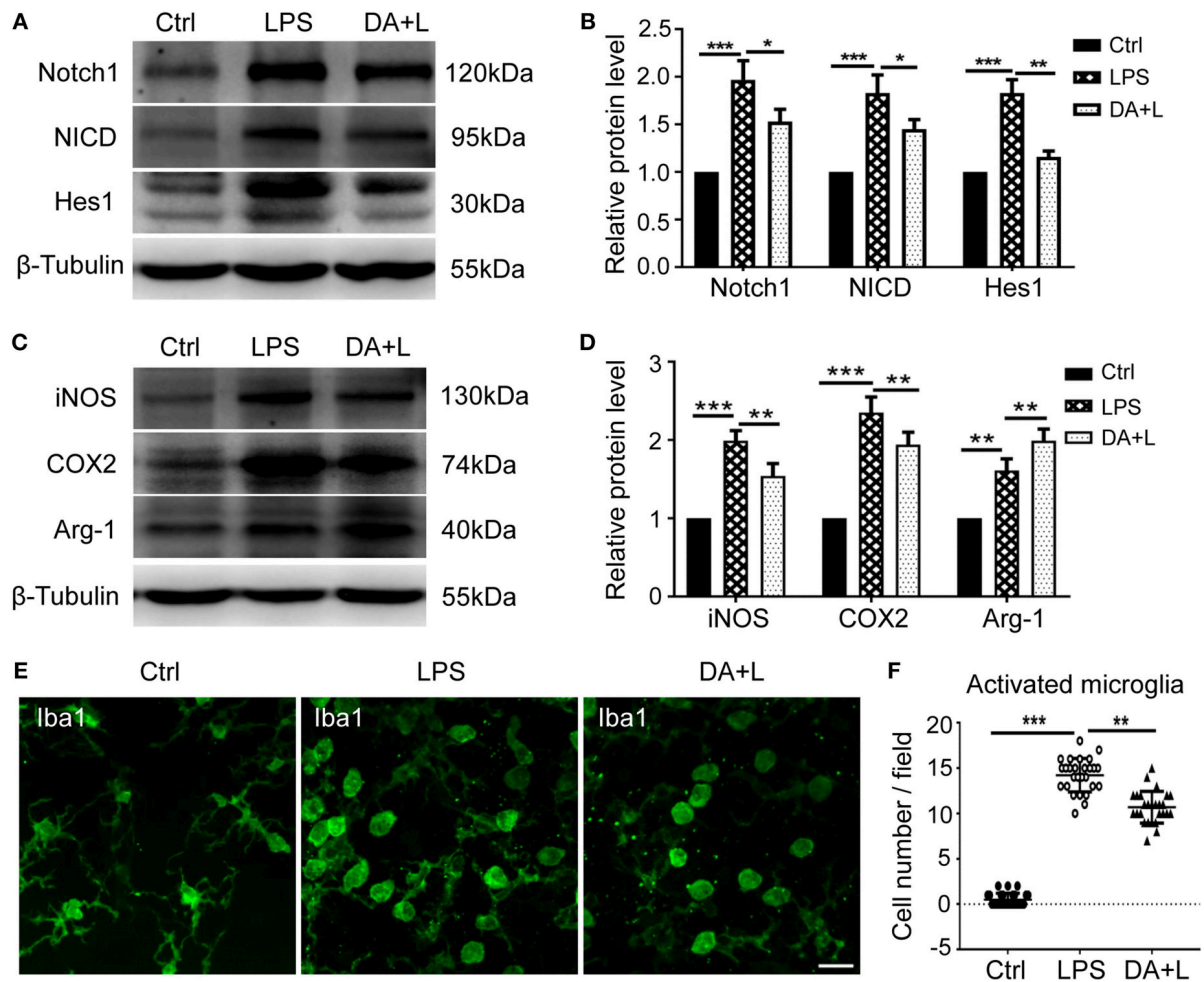
Notch1 signaling modulated activation and polarization of retinal microglia *in vivo*. **(A,B)** The protein expressions of Notch1, NICD, and Hes1 were significantly upregulated after LPS stimulation compared with the control. DAPT treatment reduced the increased expressions of Notch1, NICD, and Hes1 induced by LPS. **(C,D)** The protein expression of iNOS, COX2, and Arg-1 were markedly increased *in vivo* by LPS stimulation compared with the control. DAPT treatment suppressed the expression of iNOS and COX2, but further enhanced Arg-1 expression. **(E)** Retinal flat mounts showed that the number of amoeboid microglia in DA+L group was less than that in the LPS group. **(F)** The dot plots of activated microglia. Total 27 retinal images in each group were used for analysis. *p < 0.05, **p < 0.01, and ***p < 0.001 (one-way ANOVA). Scale bar, 40 μm. Ctrl, Control; DA+L, DAPT+LPS.

To further demonstrate the impact of Notch1 signaling on the activation and polarization of retinal microglia *in vivo*, we next investigated the protein expressions of Notch1, NICD, Hes1, iNOS, COX2, and Arg-1. The results were consistent with those of *in vitro*, further indicating that Notch1 signaling in the retina could be effectively activated by LPS and blocked by DAPT (Figures 5A,B) and inhibition of Notch1 signaling contributed to shift activated microglia from the M1 phenotype to the M2 phenotype (Figures 5C,D). In addition, the resting microglial cells typically appear as ramified morphology, while the activated microglial cells appear as amoeboid morphology. The retinal flat mounts showed that a large number of amoeboid microglia was observed after LPS injection. It was noteworthy that DAPT treatment could reduce the number of amoeboid microglia compared with the LPS group (Figures 5E,F). This finding indicated that DAPT treatment might reduce the number of activated microglia, thereby avoiding the over-activation of retinal microglia.

### DAPT Treatment Reduced the Production of Inflammatory Cytokines and Intraocular Inflammatory Cells Induced by LPS Injection

**Figure 6:**
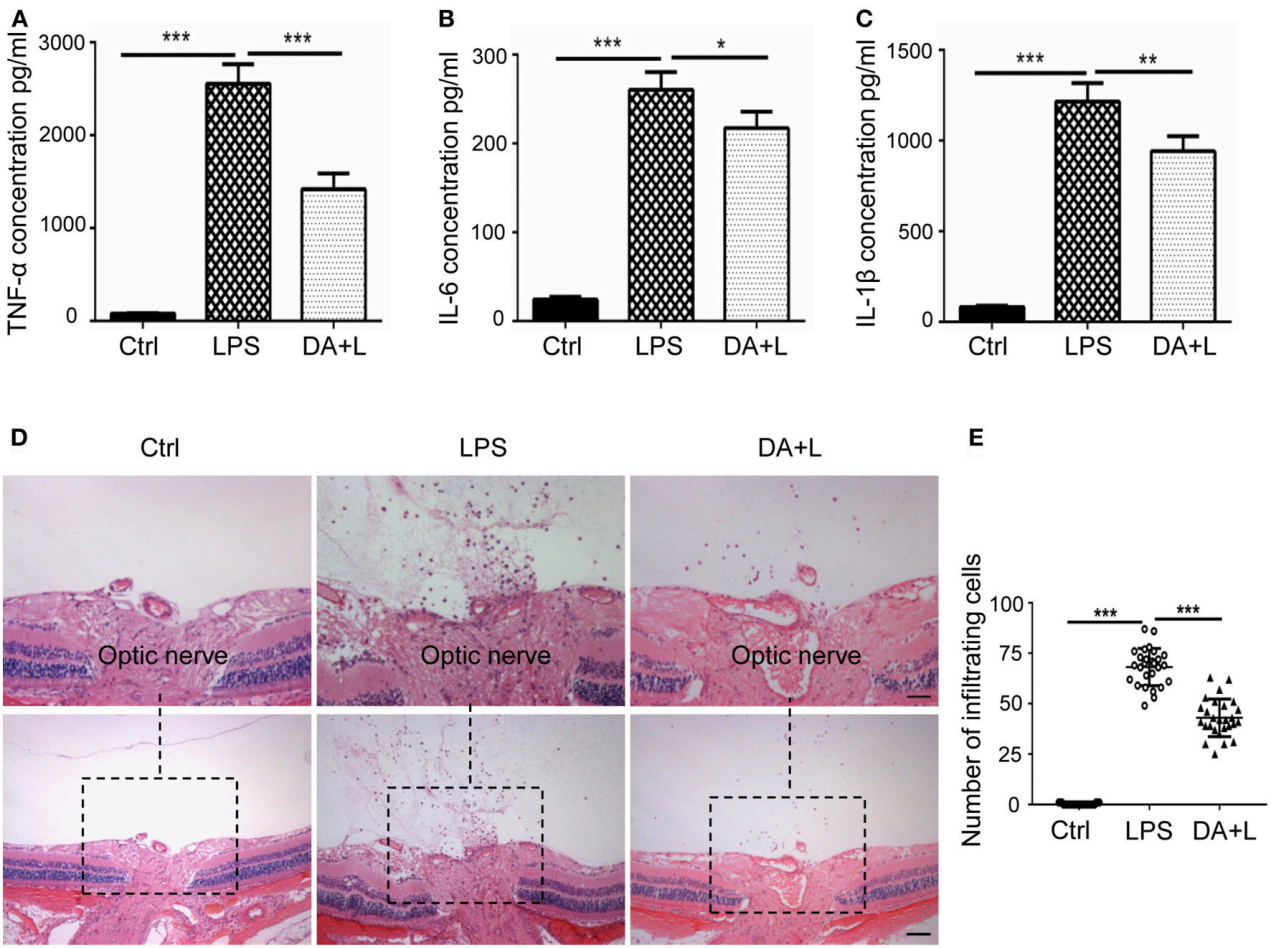
Notch1 signaling blockade reduced the production of inflammatory cytokines and the number of inflammatory cells in EIU rat eyes. **(A–C)** ELISA data showed that the concentrations of TNF-α **(A)**, IL-6 **(B)**, and IL-1β **(C)** were significantly increased after LPS stimulation in vitreous humor compared with the control. DAPT treatment reduced the production of TNF-α, IL-6, and IL-1β *in vivo*. **(D)** H&E staining showed that DAPT treatment markedly reduced the number of inflammatory infiltrating cells in vitreous cavity induced by LPS injection. **(E)** The dot plots of infiltrating cells. Total 27 retinal images in each group were used for statistical analysis. *p < 0.05, **p < 0.01, and ***p < 0.001 (one-way ANOVA). Scale bar, 100 μm. Ctrl, Control; DA+L, DAPT+LPS.

The activated microglia may release a large number of inflammatory cytokines, recruit blood-derived immune cells to infiltrate the lesions and initiate the inflammatory process. And the infiltration of inflammatory cells is an important hallmark of uveitis. Therefore, we further investigated the protein expression of TNF-α, IL-6, and IL-1β in a vitreous humor. ELISA results demonstrated that DAPT could suppress the increased expressions of inflammatory cytokines induced by LPS *in vivo* (Figures 6A–C). Additionally, we utilized vertical eye sections through the optic disc with H&E staining to evaluate the severity of an inflammatory reaction in EIU rats. A lot of inflammatory cells were observed in the posterior vitreous cavity 24 h after LPS injection. Moreover, DAPT treatment significantly reduced the number of intraocular inflammatory cells compared with the LPS group (Figures 6D,E). These findings suggested that inhibition of Notch1 signaling could reduce the production of inflammatory mediators and alleviate the infiltration of inflammatory cells in EIU rats.

### DAPT Protected RGCs From LPS-Induced Apoptosis in EIU Rats

**Figure 7:**
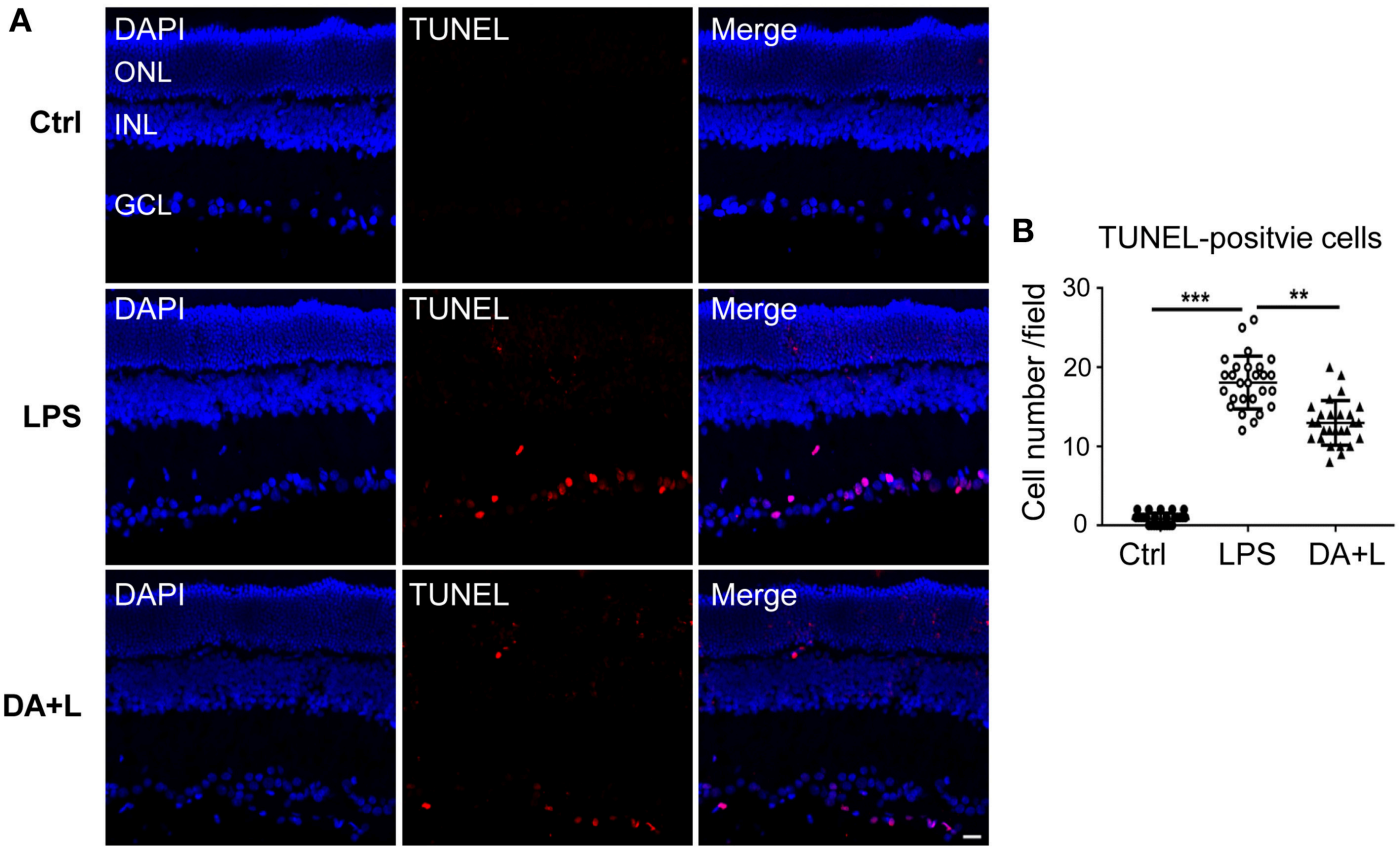
Blocking of Notch1 signaling prevented RGCs from apoptosis in EIU rats. **(A)** Confocal images showed that many TUNEL-positive cells (red) were observed in the GCL 24 h after LPS injection. DAPT treatment could remarkably reduce the number of TUNEL-positive cells (red). The nuclei were stained with DAPI (blue). Scale bar, 20 μm. **(B)** The dot plots of TUNEL-positive cells. Total 27 retinal images in each group were used for statistical analysis. **p < 0.01, ***p < 0.001 (one-way ANOVA). Ctrl, Control; DA+L, DAPT+LPS; ONL, outer nuclear layer; INL, inner nuclear layer; GCL, ganglion cell layer.

To confirm the protective effects of DAPT on RGCs after LPS injection, cell apoptosis was measured using the TUNEL kit. In the control group, there were few TUNEL-positive cells in retinal sections. Whereas, many TUNEL-positive cells were observed in the different retinal layers and mainly in the ganglion cell layer 24 h after LPS injection. Interestingly, the number of TUNEL-positive cells in the DAPT+LPS group was obviously less than that of the LPS group (Figures 7A,B). These results indicated that DAPT had a protective effect on LPS-induced RGCs' death.

## Discussion

The role of Notch1 signaling on microglia activation and polarization and microglia-induced inflammation has been explored in various cerebral inflammatory diseases (11, 14, 20–22). Few studies have investigated the role of Notch1 signaling on retinal microglia polarization and intraocular inflammation. To our knowledge, this study provided the first evidence to demonstrate that Notch1 signaling blockade switched retinal microglia polarization from the pro-inflammatory M1 phenotype to the anti-inflammatory M2 phenotype and reduced the production of inflammatory cytokines both *in vivo* and *in vitro*.

The canonical Notch signaling is initiated by Notch receptor bonding ligand. Then, the Notch receptor is cleaved by γ-secretase and the NICD is released. The NICD subsequently translocates into the nucleus and promotes the transcriptional expression of downstream target genes. The most important target genes are Hes1 and Hes5 (13, 23). Our data demonstrated that Notch1 receptor was also expressed in primary cultured retinal microglial cells. The changed expression levels of Notch1, NICD, and Hes1 indicated that Notch1 signaling could be activated by LPS and suppressed by DAPT. This finding was consistent with that in cerebral microglia, indicating that Notch1 signaling could be a modulating target for retinal microglia (13).

The molecular mechanism involved when modulating the microglia phenotype is very complex and occurs in many signal pathways (24). Understanding the molecular mechanism that defines inflammatory vs. anti-inflammatory phenotypes provides the opportunity to modulate these cellular signal pathways to control the excessive inflammation. Accumulating evidences indicate that Notch1 signaling blockade can shift the activated microglia from the M1 phenotype to the M2 phenotype, thereby alleviating the inflammation and tissue damage in cerebral diseases (8, 14, 16, 25). Our results suggested that inhibition of Notch1 signaling with DAPT could shift retinal microglia phenotype from pro-inflammatory M1 phenotype to anti-inflammatory M2 phenotype, both *in vivo* and *in vitro*. In addition, we found that Notch1 signaling blockade reduced the number of activated retinal microglia *in vivo*. This finding accorded with Zelan Wei et al. who have reported that Notch signaling blockade could markedly attenuate microglia activation induced by cerebral ischemia (14). This study suggested that Notch1 signaling had an impact on the polarization and activation of retinal microglia. However, as inflammatory mediators promote microglia activation, this could not exclude the possibility that the reduction in retinal microglia activation might be attributed to the decreased production of inflammatory cytokines.

It is well-known that over-activated microglia release excessive inflammatory cytokines or cytotoxic factors, which can exacerbate inflammatory reaction and aggravate tissue damage (15, 26, 27). The pro-inflammatory cytokines, such as TNF-α, IL-6, and IL-1β released by activated microglia, contribute to the breakdown of blood retinal barrier, the recruitment of blood-derived immune cells and neuronal damage or apoptosis (24, 28–31). In this study, we found that DAPT treatment could reduce the production of inflammatory cytokines both *in vivo* and *in vitro*, the number of intraocular infiltrating cells and apoptotic RGCs in EIU rats. These results are probably attributed to suppression of M1 activated microglia and promotion of M2 phenotype microglia. Additionally, DAPT treatment reduced the total number of activated microglia in the retina, thereby reducing the secretion of inflammatory cytokines. ERG results further suggested that DAPT treatment could attenuate the impairment of retinal function caused by LPS injection. This finding was consistent with a previous study, which indicates that inhibiting inflammation with telmisartan in EIU mice can improve retinal function (32). Of course, retinal vascular endothelial cells also express Notch1 receptor. Furthermore, the injury of retinal endothelial cells induced by LPS may lead to the breakdown of blood-retinal barrier, which may also contribute to the infiltration of inflammatory cells (33), but not initiate inflammatory reaction and mediate amplification of inflammation (34). As the resident immune cells in retina, retinal microglial cells play a pivotal role in the innate and acquired immune response to keep retinal homeostasis (5, 35). Intravitreal injection of LPS rapidly induces microglia activation, which initiates the innate immune response and inflammatory reaction (36, 37). Therefore, DAPT treatment alleviated intraocular inflammation and attenuated the RGCs apoptosis mainly through modulating retinal microglia.

As we know, amyloid beta (Aβ), a molecule within another signaling pathway of DAPT, is also involved in inflammation in the central nervous system and ocular tissues (38–40). It should not be neglected that DAPT may also have impact on the Aβ, which should be investigated in future study. In addition, our dosage regimen involved local application and intravitreal injection with a single dose DAPT to avoid or minimize the Notch-related adverse effects found in several preclinical and clinical trials (41–43). Our data suggested that intravitreal injection of DAPT at a final concentration of 200 μM was safe and effective for inhibiting Notch signaling in the eyes of LPS-induced EIU rats (data not shown).

In summary, this study demonstrated that inhibition of Notch1 signaling could ameliorate intraocular inflammation and switch retinal microglia from the M1 phenotype to the M2 phenotype, thereby contributing to attenuation of the RGCs apoptosis and the impairment of retinal function in EIU rats. Therefore, the inhibition of Notch1 signaling might be a promising strategy for the treatment of ocular inflammatory diseases.

## Author Contributions

ZC performed experiments, analyzed data, and wrote the manuscript. YY and XL designed and supervised the experiments. YL supervised the experiments and revised manuscript. BL, FD, JZ, and YH performed part of the experiments.

### Conflict of Interest Statement

The authors declare that the research was conducted in the absence of any commercial or financial relationships that could be construed as a potential conflict of interest.

## Abbreviations

DAPT: N-[N-(3, 5-difluorophenacetyl)-1-alany1-S-phenyglycine t-butyl ester
EIU: endotoxin-induced uveitis
NICD: Notch intracellular domain
iNOS: inducible nitric oxide synthase
COX2: cyclooxygenase-2
Arg-1: arginase-1
Hes1: hairy enhancer of split-1
LPS: lipopolysaccharide
Iba1: ionized calcium-binding adapter molecule 1
GAPDH: glyceraldehyde-3-phosphate dehydro-genase
TNF-α: tumor necrosis factor-α
IL-6: interleukin-6
IL-1β: interleukin-1β
ELISA: enzyme-linked immunosorbent assay
ERG: electroretinogram
TUNEL: TdT-mediated dUTP nick end labeling
RGC: retinal ganglion cell
ONL: outer nuclear layer
INL: inner nuclear layer
GCL: ganglion cell layer.
